# Quantization-Aware Hybrid Anti-Interference Receiver for Partially Connected Arrays with Low-Resolution ADCs

**DOI:** 10.3390/s26185977

**Published:** 2026-09-21

**Authors:** Dahai Ni, Chaolin Zeng, Hongbo Yin, Kun Chen, Xiangning Fan, Peng Chen

**Affiliations:** 1 School of Information Science and Engineering, Southeast University, Nanjing 210096, China; seuseayou@seu.edu.cn (D.N.); xnfan@seu.edu.cn (X.F.); 2 Yangzhou Institute of Marine Electronic Instruments, Yangzhou 225100, China; liam_research@163.com (C.Z.); yinhongbo2004@126.com (H.Y.); ckckck333@163.com (K.C.); 3 State Key Laboratory of Millimeter Waves, Southeast University, Nanjing 210096, China

**Keywords:** hybrid beamforming, interference suppression, low-resolution ADC, quantized receiver, partially connected array, manifold optimization, sparse covariance fitting

## Abstract

Partially connected hybrid analog-digital beamforming architectures substantially reduce the hardware cost and power dissipation of massive antenna arrays by routing multiple antenna elements to a single radio-frequency (RF) chain. In contested or dense electromagnetic environments, however, strong directional interference entering low-resolution analog-to-digital converters (ADCs) increases signal-dependent quantization distortion and can exceed the converter range if gain control is inadequate. To overcome this fundamental bottleneck, this paper develops a quantization-aware hybrid anti-interference receiver for partially connected architectures constrained by finite-resolution phase shifters and low-bit ADCs. The proposed receiver operates in three coordinated stages. In the spatial sensing stage, the receiver sequentially applies multiple pseudo-random analog combining configurations to collect compressed spatial observations, and reconstructs the angular power spectrum of unknown interference sources via an additive quantization noise model (AQNM)-corrected nonnegative sparse covariance fitting problem solved by the fast iterative shrinkage-thresholding algorithm (FISTA). In the analog beamforming stage, using the reconstructed interference-plus-noise covariance, subarray analog combiners are optimized directly on the complex constant-modulus manifold via Riemannian gradient descent and mapped to discrete phase states, forming analog spatial nulls to reduce interference before quantization. In the digital combining stage, a diagonally loaded robust minimum variance distortionless response (R-MVDR) combiner is formulated directly on the quantized RF domain to suppress residual interference and tolerate covariance estimation errors. Comprehensive simulations demonstrate that the proposed receiver improves output signal-to-interference-plus-noise ratio (SINR) relative to the evaluated hybrid baselines, maintaining substantial performance advantages over conventional hybrid beamforming baselines across diverse interference powers, ADC bit depths (3–8 bits), coarse phase quantization (3–8 bits), reduced RF chain counts, and limited snapshot budgets.

## 1. Introduction

Massive antenna arrays provide high spatial degrees of freedom that are essential for directional beamforming, spatial multiplexing, and interference suppression in modern wireless communication, radar, and sensing systems [1,2]. In a fully digital array, each antenna element requires a dedicated radio-frequency (RF) chain and a pair of analog-to-digital converters (ADCs), whose resolution is a design choice. As array dimensions scale up at millimeter-wave (mmWave) and sub-terahertz frequencies, deploying hundreds of high-speed transceivers incurs prohibitive hardware costs, interconnect routing complexity, and power dissipation [3]. To address these hardware bottlenecks, hybrid analog–digital architectures have emerged as an attractive solution [4,5]. By connecting a large number of antennas to a small number of RF chains through an analog phase-shifter network, hybrid architectures retain substantial array aperture gain while significantly reducing hardware overhead.

In contested or dense electromagnetic environments, hybrid array receivers must operate in the presence of strong directional interference from co-channel emitters, adjacent radar transmitters, or intentional jammers, where interference-to-noise ratios (INRs) frequently exceed 20 dB to 30 dB. In a fully digital receiver, high-dimensional spatial filtering is performed directly on uncompressed baseband signals. In contrast, a hybrid receiver must compress the *N*-dimensional antenna-domain signal into an $N_{\mathrm{RF}}$-dimensional RF-chain domain ($N_{\mathrm{RF}}\ll N$) before quantization and digital baseband processing. If strong interference is not mitigated in the analog domain before this compression, it enters the ADCs at full power. When low-resolution ADCs (e.g., 2–6 bits) are deployed to conserve power and circuit area, this high-power interference increases quantization distortion; physical clipping additionally depends on full-scale range and gain control [6,7]. Once nonlinearly distorted, the signal cannot be fully recovered by digital post-processing. Consequently, effective anti-interference in low-resolution hybrid receivers requires a joint design that coordinates analog spatial filtering, ADC dynamic range protection, and digital post-combining.

Hybrid beamforming has been extensively investigated over the past decade. Early works focused primarily on decomposing a fully digital precoder into the product of an analog beamforming matrix and a low-dimensional digital precoder, aiming to maximize spectral efficiency under sparse channel models [8,9]. To accommodate the constant-modulus constraints of analog phase shifters, techniques such as orthogonal matching pursuit (OMP) [8,10], alternating minimization [11], and Riemannian manifold optimization [11] were introduced. For partially connected architectures—where each antenna belongs to a single subarray connected to one RF chain—specialized block-diagonal algorithms have been developed to further reduce circuit layout complexity [12,13]. In addition, the effects of finite-resolution phase shifters have been addressed via discrete codebook searches or projection techniques [14], and data-driven learning methods have recently been explored to handle channel uncertainties [15,16]. Nevertheless, most existing hybrid beamforming methods focus on communication link capacity under known channel state information (CSI), whereas interference suppression requires estimating the unknown interference statistics available to the receiver.

Adaptive interference suppression in array processing is classically addressed using minimum variance distortionless response (MVDR) beamforming and its robust variants [17,18]. Directly applying these methods to hybrid arrays is challenging because the receiver cannot directly observe the full antenna-domain covariance matrix. The compressed covariance observed at the RF chains lacks the full spatial dimensionality needed to identify individual interference directions. Furthermore, the block-diagonal structure of partially connected arrays limits the spatial aperture available to each RF chain. A phase-extraction hybrid MVDR baseline computes an unconstrained digital MVDR vector and uses its phase angles to configure the analog phase shifters. However, when the optimal unconstrained weights have non-uniform amplitudes across array elements, phase-only extraction introduces substantial approximation errors. These errors deepen as phase-shifter resolution decreases, degrading the synthesized spatial nulls. Because the constant-modulus constraint is applied as an afterthought rather than during optimization, the resulting beamformer offers no guarantee on the achieved signal-to-interference-plus-noise ratio (SINR).

To reconstruct the spatial power distribution of unknown sources from compressed observations, sparse spectrum estimation and compressed sensing techniques have been widely applied [19,20]. Exploiting the spatial sparsity of directional emitters, these methods reconstruct the angular power spectrum from a reduced number of measurements. In particular, multi-configuration sensing strategies sequentially switch the analog phase-shifter network across multiple states, gathering diverse spatial projections to reconstruct the full covariance matrix in partially connected arrays [21]. However, most existing sparse recovery frameworks assume ideal, unquantized observations or model errors purely as additive white Gaussian noise. In low-resolution receivers, ADC quantization introduces non-negligible, signal-dependent distortion that systematically biases both the diagonal and off-diagonal entries of the estimated sample covariance matrix, corrupting the subsequent sparse reconstruction if left uncompensated.

To statistically characterize coarse quantization, the additive quantization noise model (AQNM) approximates the quantizer output as an attenuated signal plus uncorrelated distortion [7,22]. Related low-resolution hybrid receiver studies examine rate- and quantization-aware combining [22,23]. Analog interference mitigation also has direct precedents in beam-slicing, HERMIT, and hybrid-subarray nulling [24,25,26]. The system-level contribution here is to connect AQNM-corrected multi-configuration covariance sensing with subarray phase optimization and quantized-domain MVDR combining. The work does not introduce a new ADC or phase-shifter circuit. Its potential benefit is reduced pre-quantization interference with fewer RF chains; the costs are sensing/configuration overhead and dependence on array calibration and interference stationarity.

To bridge this gap, this paper develops a quantization-aware hybrid anti-interference receiver tailored for partially connected array architectures equipped with finite-resolution phase shifters and low-resolution ADCs. The primary contributions of this work are fourfold:

First, we establish a multi-configuration spatial sensing framework that applies *L* pseudo-random analog combining configurations to collect compressed spatial observations. By deriving a closed-form AQNM inverse covariance correction, the proposed method compensates for the covariance bias represented by the adopted AQNM and solves a regularized nonnegative sparse covariance fitting problem via FISTA, reliably estimating the angular power distribution of unknown interference and thermal noise power.

Second, rather than heuristically truncating an unconstrained solution, subarray analog combiners are optimized directly on the complex constant-modulus manifold using the reconstructed interference covariance. A Riemannian gradient descent algorithm seeks to improve the local subarray output SINR, followed by nearest-neighbor discrete phase projection, forming pre-quantization analog nulls to attenuate interference.

Third, with the analog combiner configured, a diagonally loaded robust MVDR combiner is formulated directly on the quantized RF domain. This stage cancels residual interference and reduces sensitivity to finite-snapshot covariance errors without requiring pre-quantization covariance inversion.

Finally, extensive simulations demonstrate that the proposed receiver improves SINR over the evaluated hybrid baselines across the reported operational settings, maintaining robust advantages over conventional hybrid beamforming baselines across diverse interference powers, ADC bit depths (3–8 bits), coarse phase quantization (3–8 bits), reduced RF chain counts, and limited snapshot budgets.

Notation: Boldface lowercase and uppercase letters denote vectors and matrices, respectively. Superscripts ${\left(\cdot \right)}^{T}$, (·)*, and ${\left(\cdot \right)}^{H}$ denote transpose, complex conjugate, and conjugate transpose operations. The notation ${∥\;\cdot \;∥}_{F}$ represents the Frobenius norm, ${∥\;\cdot \;∥}_{\infty }$ is the infinity norm, tr(·) denotes the matrix trace, diag(A) extracts the main diagonal of matrix A as a vector, and Diag(v) forms a diagonal matrix from vector v. The symbol ⊙ stands for the element-wise Hadamard product, ∠(·) extracts the phase angle element-wise, Re{·} extracts the real part, and CN(0,Σ) denotes the circularly symmetric complex Gaussian distribution with covariance matrix Σ.

## 2. System Model and Problem Formulation

The narrowband array and output-SINR models follow standard array processing [17,18]; low-resolution covariance modeling follows the Bussgang/AQNM literature [7,22]. The formulation combines these established ingredients with partially connected, discrete-phase constraints. Section 3 gives a sequential solution, without claiming global optimality of the joint problem.

### 2.1. Hardware-Constrained Partially Connected Array Architecture

Consider a narrowband array receiver equipped with an *N*-element uniform linear array (ULA) operating in the presence of an intended desired signal and $N_{J}$ unknown directional interference sources. The spatial direction of the desired signal is assumed to be known via prior direction-finding or initial beam-training procedures. In contrast, the number, directions, powers, and temporal waveforms of the interferers are unknown a priori.

**Figure 1:**
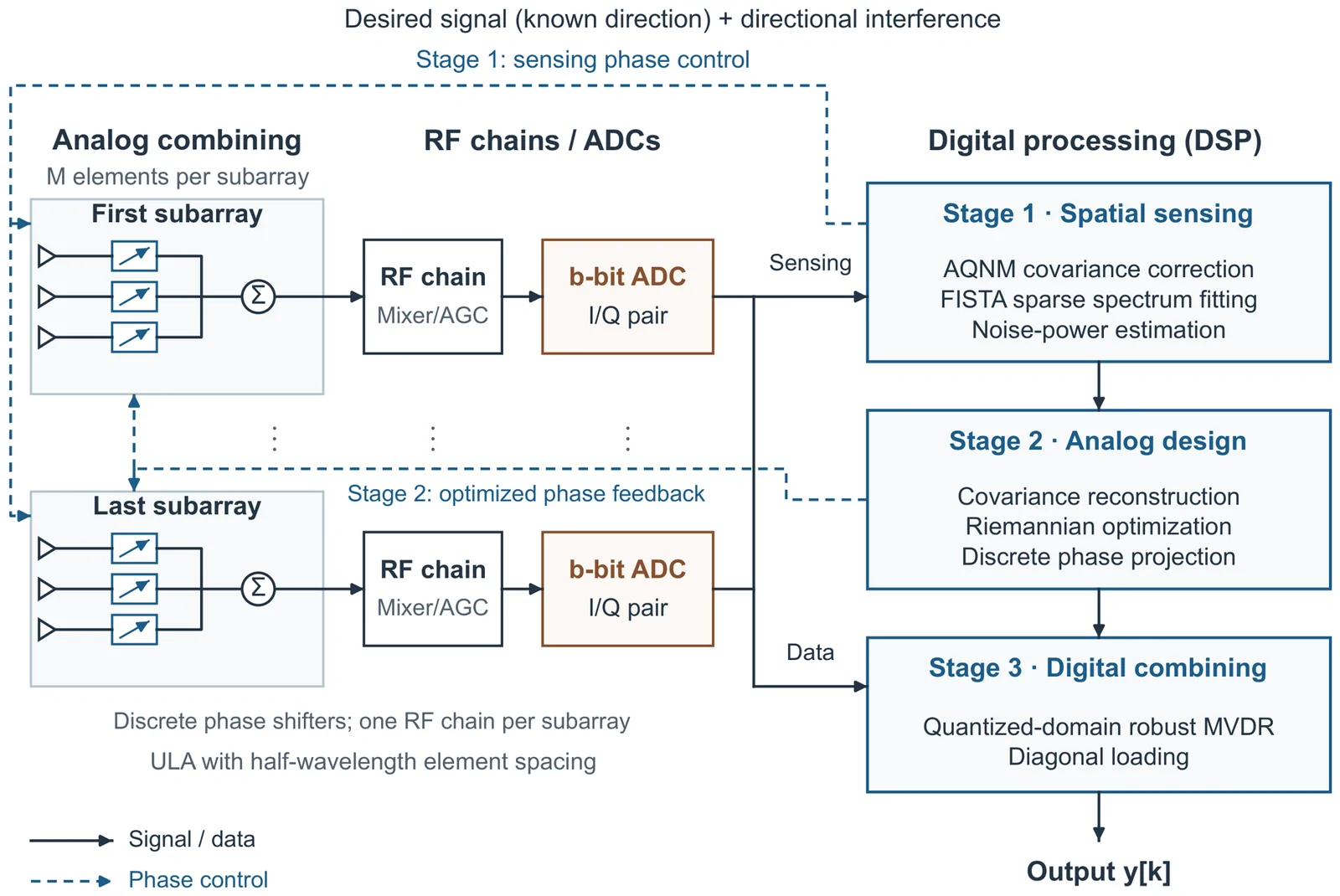
Architecture of the partially connected hybrid receiver with finite-resolution phase shifters and low-resolution ADCs. The enlarged labels distinguish sensing control from analog-weight feedback.

To strike a practical balance between performance, power dissipation, and hardware cost, a partially connected hybrid analog–digital architecture is employed. As illustrated in Figure 1, the *N* antennas are partitioned into $N_{\mathrm{RF}}$ non-overlapping uniform subarrays, with each subarray dedicated to a single RF chain. The number of antenna elements per subarray is

(1) $$M=\frac{N}{N_{\mathrm{RF}}},$$

where *N* is assumed to be an integer multiple of $N_{\mathrm{RF}}$. Each RF chain is terminated with a pair of *b*-bit ADCs for in-phase and quadrature baseband sampling. Analog spatial combining within each subarray is implemented via a network of discrete phase shifters with $B_{\mathrm{PS}}$ bits of resolution. Thus, the receiver operates under three concurrent hardware constraints: A limited number of RF chains ($N_{\mathrm{RF}}\ll N$), discrete phase-shifter states, and low ADC quantization resolution.

The partially connected analog combining matrix $W_{\mathrm{RF}}\in C^{N\times N_{\mathrm{RF}}}$ exhibits a block-diagonal structure:

(2) $$W_{\mathrm{RF}}=\mathrm{blkdiag}\left\{w_{1},w_{2},\ldots ,w_{N_{\mathrm{RF}}}\right\},$$

where $w_{i}\in C^{M\times 1}$ is the analog combining vector for the *i*-th subarray ($i=1,\ldots ,N_{\mathrm{RF}}$). With $B_{\mathrm{PS}}$ bits of phase control, each entry of $w_{i}$ is restricted to the discrete constant-modulus set

(3) $$P_{B_{\mathrm{PS}}}=\left\{\frac{1}{\sqrt{M}}\exp\left(j\frac{2\pi q}{2^{B_{\mathrm{PS}}}}\right)\;|\;q=0,1,\ldots ,2^{B_{\mathrm{PS}}}-1\right\}.$$

This ensures ${\left[w_{i}\right]}_{m}\in P_{B_{\mathrm{PS}}}$ for m=1,…,M, satisfying the subarray normalization ${∥w_{i}∥}_{2}^{2}=1$. Owing to the non-overlapping subarray geometry, the full analog matrix satisfies the semi-unitary property:

(4) $$W_{\mathrm{RF}}^{H}W_{\mathrm{RF}}=I_{N_{\mathrm{RF}}}.$$

### 2.2. Narrowband Received Signal and Covariance Model

Let $x\left[k\right]\in C^{N\times 1}$ denote the received signal vector at the antenna aperture at discrete snapshot index *k*:

(5) $$x\left[k\right]=\sqrt{P_{s}}a\left(u_{s}\right)s\left[k\right]+\sum_{j=1}^{N_{J}}\sqrt{P_{j}}a\left(u_{j}\right)i_{j}\left[k\right]+n\left[k\right],$$

where s[k]∈C and $i_{j}\left[k\right]\in C$ represent the normalized baseband waveforms of the desired signal and the *j*-th interference source, respectively; $P_{s}$ and $P_{j}$ are their respective received powers at the antenna aperture and $n\left[k\right]∼\mathrm{CN}\left(0,\sigma_{n}^{2}I_{N}\right)$ is the additive white Gaussian thermal noise vector. The spatial frequency *u* is defined as

(6) u=sinθ,u∈[−1,1],

where θ∈[−π/2,π/2] is the physical angle of arrival. For a half-wavelength-spaced ULA, the normalized array steering vector $a\left(u\right)\in C^{N\times 1}$ satisfies ${∥a\left(u\right)∥}_{2}=1$ and is given by

(7) $$a(u)=\frac{1}{\sqrt{N}}{\left[1,e^{-j\pi u},\ldots ,e^{-j\pi (N-1)u}\right]}^{T}.$$

The desired source waveform s[k] and interference waveforms ${\left\{i_{j}\left[k\right]\right\}}_{j=1}^{N_{J}}$ are assumed to be mutually uncorrelated zero-mean stationary processes satisfying

(8) $$E\left\{{\left|s\left[k\right]\right|}^{2}\right\}=1,\;E\left\{|i_{j}{\left[k\right]|}^{2}\right\}=1,$$

(9) $$E\left\{s\left[k\right]i_{j}^{*}\left[k\right]\right\}=0,\;E\left\{i_{j}\left[k\right]i_{\ell }^{*}\left[k\right]\right\}=0\;\left(\forall j\neq \ell \right).$$

The antenna-domain covariance matrix $R_{xx}=E\left\{x\left[k\right]x^{H}\left[k\right]\right\}\in C^{N\times N}$ is, therefore,

(10) $$R_{xx}=P_{s}a(u_{s})a^{H}(u_{s})+\sum_{j=1}^{N_{J}}P_{j}a(u_{j})a^{H}(u_{j})+\sigma_{n}^{2}I_{N}.$$

Prior to ADC quantization, the signal vector at the RF chain outputs is

(11) $$z\left[k\right]=W_{\mathrm{RF}}^{H}x\left[k\right]\in C^{N_{\mathrm{RF}}\times 1},$$

and its unquantized covariance matrix is

(12) $$R_{zz}=E\left\{z\left[k\right]z^{H}\left[k\right]\right\}=W_{\mathrm{RF}}^{H}R_{xx}W_{\mathrm{RF}}.$$

### 2.3. Low-Resolution ADC and AQNM Quantization Distortion Model

Each RF chain output is quantized by a pair of *b*-bit ADCs. An automatic gain control (AGC) unit adjusts the signal level to match the linear operating range of the converters. For multi-bit converters (typically b≥2), the quantization process is modeled via the AQNM [7,22]:

(13) $$\tilde{z}\left[k\right]=\alpha_{b}z\left[k\right]+q\left[k\right],$$

where $\alpha_{b}=1-\rho_{b}$ is the linear quantization gain and $q\left[k\right]\in C^{N_{\mathrm{RF}}\times 1}$ is the additive quantization noise vector, uncorrelated with z[k]. The distortion factor $\rho_{b}$ uses the Gaussian-input high-resolution approximation [6,22]

(14) $$\rho_{b}\approx \frac{\pi \sqrt{3}}{2}2^{-2b}.$$

With the additional diagonal distortion-covariance approximation, the covariance matrix of the quantized signal $\tilde{z}\left[k\right]$ is given by

(15) $$R_{\tilde{z}\tilde{z}}=E\left\{\tilde{z}\left[k\right]{\tilde{z}}^{H}\left[k\right]\right\}\approx \alpha_{b}^{2}R_{zz}+\alpha_{b}\left(1-\alpha_{b}\right)\mathrm{Diag}\left(\mathrm{diag}(R_{zz})\right).$$

Examining the main diagonal of Equation (15) yields

(16) $$\mathrm{diag}\left(R_{\tilde{z}\tilde{z}}\right)\approx \alpha_{b}\mathrm{diag}\left(R_{zz}\right).$$

Substituting Equation (16) into Equation (15) gives an algebraic inverse within the adopted AQNM covariance approximation:

(17) $$R_{zz}\approx \frac{1}{\alpha_{b}^{2}}\left[R_{\tilde{z}\tilde{z}}-\left(1-\alpha_{b}\right)\mathrm{Diag}\left(\mathrm{diag}(R_{\tilde{z}\tilde{z}})\right)\right].$$

Equation (17) allows the receiver to obtain an AQNM-corrected estimate of the compressed covariance $R_{zz}$ from quantized observations during the spatial sensing stage.

### 2.4. Joint Hybrid Anti-Interference Problem Formulation

During data reception, the beamformed receiver output is formed via a digital baseband combiner $w_{\mathrm{BB}}\in C^{N_{\mathrm{RF}}\times 1}$:

(18) $$y\left[k\right]=w_{\mathrm{BB}}^{H}\tilde{z}\left[k\right]=w_{\mathrm{BB}}^{H}\left(\alpha_{b}W_{\mathrm{RF}}^{H}x\left[k\right]+q\left[k\right]\right).$$

The overall design objective is to maximize the output SINR subject to the partially connected and discrete phase-shifter constraints:

(19) $$\begin{array}{cc} \max_{W_{\mathrm{RF}},w_{\mathrm{BB}}} & \frac{P_{s}{\left|w_{\mathrm{BB}}^{H}W_{\mathrm{RF}}^{H}a\left(u_{s}\right)\right|}^{2}}{w_{\mathrm{BB}}^{H}W_{\mathrm{RF}}^{H}\left(\sum_{j=1}^{N_{J}}P_{j}a\left(u_{j}\right)a^{H}\left(u_{j}\right)+\sigma_{n}^{2}I_{N}\right)W_{\mathrm{RF}}w_{\mathrm{BB}}+\frac{1}{\alpha_{b}^{2}}w_{\mathrm{BB}}^{H}R_{qq}w_{\mathrm{BB}}}\\ s.t. & W_{\mathrm{RF}}=\mathrm{blkdiag}\left\{w_{1},\ldots ,w_{N_{\mathrm{RF}}}\right\},\;{\left[w_{i}\right]}_{m}\in P_{B_{\mathrm{PS}}}. \end{array}$$

Directly solving Problem Equation (19) presents four coupled difficulties. First, the full antenna-domain covariance $R_{xx}$ is unobservable through the $N_{\mathrm{RF}}$ compressed RF chains. Second, the analog combining matrix $W_{\mathrm{RF}}$ is governed by discrete constant-modulus constraints in $P_{B_{\mathrm{PS}}}$. Third, strong interference entering the ADC front-end inflates the effective quantization noise covariance $R_{qq}$. Finally, the analog combiner $W_{\mathrm{RF}}$ and digital combiner $w_{\mathrm{BB}}$ are non-convexly coupled through the dimension-reduced channel. To overcome these challenges, we decouple Problem Equation (19) into a structured three-stage framework, detailed in Section 3.

## 3. Proposed Quantization-Aware Hybrid Anti-Interference Method

The proposed anti-interference framework operates in three sequential stages: Stage 1 performs quantization-aware multi-configuration spatial sensing and FISTA sparse spectrum estimation; Stage 2 executes interference covariance reconstruction and Riemannian manifold optimization on the constant-modulus manifold with discrete phase projection and Stage 3 implements quantized-domain robust MVDR baseband combining.

### 3.1. Stage 1: Quantization-Aware Multi-Configuration Spatial Sensing

Because $N_{\mathrm{RF}}\ll N$, a single partially connected combiner cannot observe the full antenna-domain spatial subspace. To overcome this limitation, the receiver sequentially applies *L* distinct analog combining configurations:

(20) $$\left\{W_{\mathrm{RF}}^{\left(1\right)},W_{\mathrm{RF}}^{\left(2\right)},\ldots ,W_{\mathrm{RF}}^{\left(L\right)}\right\},$$

where each $W_{\mathrm{RF}}^{\left(l\right)}$ satisfies Equations (2) and (3), with phase states drawn pseudo-randomly to ensure diverse spatial projections.

For the *l*-th configuration (l=1,…,L), the receiver collects $K_{l}$ quantized snapshots ${\left\{{\tilde{z}}^{\left(l\right)}\left[k\right]\right\}}_{k=1}^{K_{l}}$ and forms the sample covariance:

(21) $${\hat{R}}_{\tilde{z}\tilde{z}}^{\left(l\right)}=\frac{1}{K_{l}}\sum_{k=1}^{K_{l}}{\tilde{z}}^{\left(l\right)}\left[k\right]{\left({\tilde{z}}^{\left(l\right)}\left[k\right]\right)}^{H}.$$

Applying the AQNM inverse correction Equation (17) to ${\hat{R}}_{\tilde{z}\tilde{z}}^{\left(l\right)}$ compensates for the modeled covariance bias, yielding an estimate of the pre-quantization covariance:

(22) $${\hat{R}}_{zz}^{\left(l\right)}=\frac{1}{\alpha_{b}^{2}}\left[{\hat{R}}_{\tilde{z}\tilde{z}}^{\left(l\right)}-\left(1-\alpha_{b}\right)\mathrm{Diag}\left(\mathrm{diag}({\hat{R}}_{\tilde{z}\tilde{z}}^{\left(l\right)})\right)\right]\approx {\left(W_{\mathrm{RF}}^{\left(l\right)}\right)}^{H}R_{xx}W_{\mathrm{RF}}^{\left(l\right)}.$$

To estimate the interference angular distribution from ${\left\{{\hat{R}}_{zz}^{\left(l\right)}\right\}}_{l=1}^{L}$, we use *G* candidate angular directions and their corresponding spatial frequencies:

(23) $$U={\left\{\sin\theta_{g}\right\}}_{g=1}^{G},\;\theta_{g}=-90^{{}^{\circ}}+\frac{180^{{}^{\circ}}\left(g-1\right)}{G-1},\;g=1,\ldots ,G.$$

The corresponding spatial steering dictionary is $A_{U}=\left[a\left(u_{1}\right),\ldots ,a\left(u_{G}\right)\right]\in C^{N\times G}$. Under the uncorrelated-source model, the antenna covariance is parameterized by a nonnegative angular power vector $p={\left[p_{1},\ldots ,p_{G}\right]}^{T}⪰0$:

(24) $$R_{xx}\left(p,\sigma_{n}^{2}\right)\approx A_{U}\mathrm{Diag}\left(p\right)A_{U}^{H}+\sigma_{n}^{2}I_{N}.$$

For the *l*-th sensing configuration, the compressed steering atom for grid index *g* is

(25) $$b_{g}^{\left(l\right)}={\left(W_{\mathrm{RF}}^{\left(l\right)}\right)}^{H}a\left(u_{g}\right)\in C^{N_{\mathrm{RF}}\times 1},\;B_{g}^{\left(l\right)}=b_{g}^{\left(l\right)}{\left(b_{g}^{\left(l\right)}\right)}^{H}\in C^{N_{\mathrm{RF}}\times N_{\mathrm{RF}}}.$$

Using the semi-unitary property Equation (4), the predicted compressed covariance is

(26) $$\Phi^{\left(l\right)}\left(p,\sigma_{n}^{2}\right)=\sum_{g=1}^{G}p_{g}B_{g}^{\left(l\right)}+\sigma_{n}^{2}I_{N_{\mathrm{RF}}}.$$

Because directional sources occupy only a small subset of angular bins, the power vector p is naturally sparse. We formulate the joint estimation of p and $\sigma_{n}^{2}$ as a regularized non-negative least-squares covariance fitting problem:

(27) $$\begin{array}{cc} \min_{p⪰0,\;\sigma_{n}^{2}\geq 0} & \frac{1}{2}\sum_{l=1}^{L}{∥{\hat{R}}_{zz}^{\left(l\right)}-\Phi^{\left(l\right)}\left(p,\sigma_{n}^{2}\right)∥}_{F}^{2}+\lambda_{p}1^{T}p, \end{array}$$

where $\lambda_{p}>0$ is a sparsity-promoting regularization parameter, and $1^{T}p={∥p∥}_{1}$ holds due to the non-negativity constraint p⪰0. Problem Equation (27) is globally convex.

We solve Equation (27) using FISTA with backtracking line search [27]. Let $f\left(p,\sigma_{n}^{2}\right)=\frac{1}{2}\sum_{l=1}^{L}{∥E^{\left(l\right)}∥}_{F}^{2}$, where the residual matrix is $E^{\left(l\right)}=\Phi^{\left(l\right)}\left(p,\sigma_{n}^{2}\right)-{\hat{R}}_{zz}^{\left(l\right)}$. The partial derivatives with respect to $p_{g}$ and $\sigma_{n}^{2}$ are

(28) ∂f∂pg=Re∑l=1LtrBg(l)HE(l),g=1,…,G,

(29) ∂f∂σn2=Re∑l=1LtrE(l).

Let $y^{\left(r\right)}$ and $\nu^{\left(r\right)}$ denote the extrapolated search points at iteration *r*. Given a step size $\eta_{r}>0$, the non-negative proximal updates are

(30) $$p_{g}^{\left(r+1\right)}=\max\left\{0,\;y_{g}^{\left(r\right)}-\eta_{r}{\left.\frac{\partial f}{\partial p_{g}}\right|}_{\left(y^{\left(r\right)},\nu^{\left(r\right)}\right)}-\eta_{r}\lambda_{p}\right\},$$

(31) $${\left(\sigma_{n}^{2}\right)}^{\left(r+1\right)}=\max\left\{0,\;\nu^{\left(r\right)}-\eta_{r}{\left.\frac{\partial f}{\partial \sigma_{n}^{2}}\right|}_{\left(y^{\left(r\right)},\nu^{\left(r\right)}\right)}\right\}.$$

The extrapolation momentum updates follow the standard FISTA acceleration rule:

(32) $$t_{r+1}=\frac{1+\sqrt{1+4t_{r}^{2}}}{2},\;t_{0}=1,$$

(33) $$y^{\left(r+1\right)}=p^{\left(r+1\right)}+\frac{t_{r}-1}{t_{r+1}}\left(p^{\left(r+1\right)}-p^{\left(r\right)}\right),$$

(34) $$\nu^{\left(r+1\right)}={\left(\sigma_{n}^{2}\right)}^{\left(r+1\right)}+\frac{t_{r}-1}{t_{r+1}}\left({\left(\sigma_{n}^{2}\right)}^{\left(r+1\right)}-{\left(\sigma_{n}^{2}\right)}^{\left(r\right)}\right).$$

The step size $\eta_{r}$ is determined via backtracking line search. The iteration terminates when the relative change in the cost function drops below a preset threshold $\varepsilon_{p}$:

(35) $$\frac{|F^{\left(r+1\right)}-F^{\left(r\right)}|}{\max\{F^{\left(r\right)},\varepsilon_{0}\}}\leq \varepsilon_{p}.$$

The regularization parameter is set for each covariance-fitting data set as $\lambda_{p}=\kappa_{p}{∥\Psi^{H}r∥}_{\infty }$, where r stacks the vectorized observations ${\left\{{\hat{R}}_{zz}^{\left(l\right)}\right\}}_{l=1}^{L}$, Ψ stacks the corresponding vectorized atoms $\left\{B_{g}^{\left(l\right)}\right\}$, and $\kappa_{p}$ is a normalized scale factor.

### 3.2. Stage 2: Covariance Reconstruction and Manifold-Based Analog Beamforming

Once the estimated power spectrum $\hat{p}$ and noise power ${\hat{\sigma }}_{n}^{2}$ are obtained, the receiver reconstructs the full-dimensional interference-plus-noise covariance matrix. Because the desired signal angle $u_{s}$ is known, we define an angular protection sector around $u_{s}$:

(36) $$G_{s}=\left\{g\in \left\{1,\ldots ,G\right\}\;|\;|u_{g}-u_{s}|\leq \Delta_{u}\right\},$$

where $\Delta_{u}>0$ matches the main-lobe width of the array. The set of grid indices associated with potential interferers is $G_{J}=\left\{1,\ldots ,G\right\}\setminus G_{s}$. The full antenna-domain interference-plus-noise covariance matrix is reconstructed as

(37) $${\hat{R}}_{i+n}=\sum_{g\in G_{J}}{\hat{p}}_{g}a\left(u_{g}\right)a^{H}\left(u_{g}\right)+{\hat{\sigma }}_{n}^{2}I_{N}.$$

For the *i*-th subarray ($i=1,\ldots ,N_{\mathrm{RF}}$), let $S_{i}\in {\left\{0,1\right\}}^{M\times N}$ denote the antenna selection matrix that extracts the *M* elements of subarray *i*. The local desired steering vector is $a_{s,i}=S_{i}a\left(u_{s}\right)\in C^{M\times 1}$, and the corresponding local interference-plus-noise covariance matrix is

(38) $${\hat{R}}_{i+n,i}=S_{i}{\hat{R}}_{i+n}S_{i}^{H}+\mu_{a,i}I_{M},$$

where $\mu_{a,i}=\kappa_{a}\frac{\mathrm{tr}(S_{i}{\hat{R}}_{i+n}S_{i}^{H})}{M}$ is a diagonal loading term with $\kappa_{a}>0$ that improves numerical conditioning and reduces sensitivity to covariance perturbations.

To suppress dominant interference in the analog domain before ADC quantization, each subarray beamformer $w_{i}\in C^{M\times 1}$ is optimized using ${\hat{R}}_{i+n,i}$. Instead of deriving an unconstrained MVDR solution and truncating its amplitudes, we formulate the optimization directly on the complex constant-modulus manifold:

(39) $$M=\left\{w_{i}\in C^{M}\;|\;\left|{\left[w_{i}\right]}_{m}\right|=\frac{1}{\sqrt{M}},\;m=1,\ldots ,M\right\}.$$

Optimizing the estimated, loaded local SINR criterion amounts to minimizing the ratio of interference-plus-noise output power to desired-signal gain:

(40) $$\min_{w_{i}\in M}\;f\left(w_{i}\right)=\frac{w_{i}^{H}{\hat{R}}_{i+n,i}w_{i}}{{\left|w_{i}^{H}a_{s,i}\right|}^{2}}.$$

Let $R_{i}={\hat{R}}_{i+n,i}$ and $a_{i}=a_{s,i}$. Under the real inner product Re(uHv), the Euclidean gradient is $\nabla f=2\partial f/\partial w_{i}^{*}$, given by

(41) $$\nabla f\left(w_{i}\right)=\frac{2{\left|w_{i}^{H}a_{i}\right|}^{2}R_{i}w_{i}-2\left(w_{i}^{H}R_{i}w_{i}\right)a_{i}a_{i}^{H}w_{i}}{{\left|w_{i}^{H}a_{i}\right|}^{4}}.$$

Projecting $\nabla f(w_{i})$ onto the tangent space $T_{w_{i}}M$ of the constant-modulus manifold yields the Riemannian gradient:

(42) gradf(wi)=∇f(wi)−MRe∇f(wi)⊙wi*⊙wi.

The factor *M* follows from the normalized radius $|{\left[w_{i}\right]}_{m}|=1/\sqrt{M}$ and ensures tangency.

The beamforming vector is iteratively updated along the Riemannian steepest descent direction:

(43) $$w_{i}^{\left(t+1\right)}=\mathrm{Retr}\left(w_{i}^{\left(t\right)}-\beta_{t}\mathrm{grad}f\left(w_{i}^{\left(t\right)}\right)\right),$$

where $\beta_{t}>0$ is the step size and Retr(·) is the retraction operator defined element-wise as

(44) $$\mathrm{Retr}(v)=\frac{1}{\sqrt{M}}\exp\left(j∠(v)\right).$$

The iteration continues until the relative change in objective value satisfies

(45) $$\frac{|f\left(w_{i}^{\left(t+1\right)}\right)-f\left(w_{i}^{\left(t\right)}\right)|}{\max\{f\left(w_{i}^{\left(t\right)}\right),\varepsilon \}}\leq \varepsilon_{m},$$

where $\varepsilon_{m}$ is the convergence tolerance and ε>0 prevents division by zero.

Upon convergence to a continuous-phase solution $w_{i}$, each phase coefficient is mapped to the closest discrete state in $P_{B_{\mathrm{PS}}}$:

(46) $${\left[w_{i}^{★}\right]}_{m}=\arg\min_{\zeta \in P_{B_{\mathrm{PS}}}}\left|{\left[w_{i}\right]}_{m}-\zeta \right|,\;m=1,\ldots ,M.$$

The aggregate analog combining matrix for data reception is assembled as

(47) $$W_{\mathrm{RF}}^{★}=\mathrm{blkdiag}\left\{w_{1}^{★},w_{2}^{★},\ldots ,w_{N_{\mathrm{RF}}}^{★}\right\}.$$

### 3.3. Stage 3: Quantized-Domain Robust Digital MVDR Combining

With the analog combiner fixed to $W_{\mathrm{RF}}^{★}$, the receiver enters the data reception stage. In this stage, $K_{d}$ quantized baseband snapshots ${\left\{{\tilde{z}}^{★}\left[k\right]\right\}}_{k=1}^{K_{d}}$ are collected at the RF chain outputs:

(48) $${\tilde{z}}^{★}\left[k\right]=\alpha_{b}{\left(W_{\mathrm{RF}}^{★}\right)}^{H}x\left[k\right]+q\left[k\right].$$

The sample covariance matrix of the quantized observations is computed as

(49) $${\hat{R}}_{\tilde{z}}=\frac{1}{K_{d}}\sum_{k=1}^{K_{d}}{\tilde{z}}^{★}\left[k\right]{\left({\tilde{z}}^{★}\left[k\right]\right)}^{H}\in C^{N_{\mathrm{RF}}\times N_{\mathrm{RF}}}.$$

Unlike the sensing stage, no AQNM inversion is applied to ${\hat{R}}_{\tilde{z}}$. Because the baseband digital processor operates directly on the actual quantized data stream ${\tilde{z}}^{★}\left[k\right]$, the digital MVDR weights must be optimized against the true quantized-domain statistics rather than the unquantized covariance.

The effective desired steering vector at the RF chain outputs is

(50) $$c_{s}={\left(W_{\mathrm{RF}}^{★}\right)}^{H}a\left(u_{s}\right)\in C^{N_{\mathrm{RF}}\times 1}.$$

To reduce sensitivity to finite-sample fluctuations and improve covariance conditioning, we apply diagonal loading:

(51) $${\hat{R}}_{d}={\hat{R}}_{\tilde{z}}+\delta_{d}I_{N_{\mathrm{RF}}},\;\delta_{d}=\kappa_{d}\frac{\mathrm{tr}\left({\hat{R}}_{\tilde{z}}\right)}{N_{\mathrm{RF}}},$$

where $\kappa_{d}>0$ is a dimensionless loading coefficient (typically $10^{-2}$ to $10^{-1}$). The optimal digital R-MVDR combiner is obtained in closed form:

(52) $$w_{\mathrm{BB}}^{★}=\frac{{\hat{R}}_{d}^{-1}c_{s}}{c_{s}^{H}{\hat{R}}_{d}^{-1}c_{s}}.$$

The final beamformed scalar output is

(53) $$y\left[k\right]={\left(w_{\mathrm{BB}}^{★}\right)}^{H}{\tilde{z}}^{★}\left[k\right].$$

### 3.4. Algorithm Workflow, Computational Complexity, and Hardware Feasibility

The overall execution procedure is summarized in Algorithm 1.
**Algorithm 1** Proposed Quantization-Aware Hybrid Anti-Interference Algorithm**Require:** Sensing combiners ${\left\{W_{\mathrm{RF}}^{\left(l\right)}\right\}}_{l=1}^{L}$, quantized observations ${\left\{{\hat{R}}_{\tilde{z}\tilde{z}}^{\left(l\right)}\right\}}_{l=1}^{L}$, desired direction $u_{s}$, ADC bit depth *b*, phase resolution $B_{\mathrm{PS}}$.
**Ensure:** Analog combining matrix $W_{\mathrm{RF}}^{★}$, digital combining vector $w_{\mathrm{BB}}^{★}$. 1:// Stage 1: Spatial Sensing and Sparse Recovery2:Compute quantization gain factor $\alpha_{b}=1-\frac{\pi \sqrt{3}}{2}2^{-2b}$3:Apply AQNM inverse correction Equation (22) to obtain corrected pre-quantization covariance estimates ${\left\{{\hat{R}}_{zz}^{\left(l\right)}\right\}}_{l=1}^{L}$.4:Construct compressed spatial atoms $\left\{B_{g}^{\left(l\right)}\right\}$ on grid U via Equation (25).5:Estimate angular power spectrum $\hat{p}$ and noise power ${\hat{\sigma }}_{n}^{2}$ by solving Equation (27) via FISTA.6:// Stage 2: Manifold-Based Analog Optimization7:Mask out the desired-signal sector $G_{s}$ and reconstruct the antenna-domain interference-plus-noise covariance ${\hat{R}}_{i+n}$ via Equation (37).8:**for** i=1**to**$N_{\mathrm{RF}}$**do**9:      Extract subarray covariance ${\hat{R}}_{i+n,i}$ via Equation (38) and target steering vector $a_{s,i}$ = $S_{i}a\left(u_{s}\right)$.10:      Initialize $w_{i}^{\left(0\right)}=\frac{1}{\sqrt{M}}\exp\left(j∠\left(a_{s,i}\right)\right)$.11:     Optimize $w_{i}$ on constant-modulus manifold M using Riemannian gradient updates Equation (43) until convergence.12:     Quantize phase coefficients to discrete set $P_{B_{\mathrm{PS}}}$ via Equation (46) to yield $w_{i}^{★}$.13:**end for**14:Assemble global analog combiner $W_{\mathrm{RF}}^{★}=\mathrm{blkdiag}\left\{w_{1}^{★},\ldots ,w_{N_{\mathrm{RF}}}^{★}\right\}$.15:// Stage 3: Quantized-Domain Digital Combining16:Collect $K_{d}$ quantized snapshots under $W_{\mathrm{RF}}^{★}$ and compute sample covariance ${\hat{R}}_{\tilde{z}}$ via Equation (49).17:Apply diagonal loading Equation (51) and evaluate digital R-MVDR weight vector $w_{\mathrm{BB}}^{★}$ via Equation (52).18:**return** $W_{\mathrm{RF}}^{★}$, $w_{\mathrm{BB}}^{★}$.


#### 3.4.1. Computational Complexity Analysis

The computational complexity of the proposed framework spans three modular operations. For the sparse angular spectrum estimation in Stage 1, evaluating the residuals ${\left\{E^{\left(l\right)}\right\}}_{l=1}^{L}$ and computing gradients Equation (28) and Equation (29) requires $O(LGN_{\mathrm{RF}}^{2})$ operations per FISTA iteration, yielding an overall complexity of $O(T_{p}LGN_{\mathrm{RF}}^{2})$ across $T_{p}$ iterations. For the manifold analog optimization in Stage 2, optimization is executed independently across the $N_{\mathrm{RF}}$ subarrays. For each *M*-element subarray, Riemannian gradient computation and retraction require $O(M^{2})$ operations per iteration, leading to an overall complexity of $O\left(T_{m}N_{\mathrm{RF}}M^{2}\right)=O\left(T_{m}NM\right)$ across $T_{m}$ iterations. For the quantized-domain digital MVDR in Stage 3, inverting the loaded $N_{\mathrm{RF}}\times N_{\mathrm{RF}}$ covariance matrix ${\hat{R}}_{d}$ requires $O(N_{\mathrm{RF}}^{3})$ operations. The digital solve is smaller than an N×N inversion; total cost also depends on sensing configurations, dictionary size and iteration counts. The primary computational burden occurs during the spatial sensing phase, whereas real-time data combining requires only low-dimensional matrix-vector multiplications.

#### 3.4.2. Hardware Implementation Feasibility

The proposed receiver architecture is well matched to practical hybrid array hardware. In terms of topology, dedicating each antenna subarray to a single RF chain reduces the required number of RF chains by a factor of *M*, reducing RF-chain count relative to a fully digital array; total receiver power also depends on the analog and control circuitry. In terms of phase control, projecting the continuous solution onto $P_{B_{\mathrm{PS}}}$ ensures that standard discrete phase shifters (such as 3-bit or 4-bit PIN diode or CMOS phase shifters) can be directly employed without requiring high-precision analog control circuitry. Finally, the spatial sensing stage relies on phase-shifter configuration switching commanded via digital control registers, while baseband algorithms operate on low-dimensional $N_{\mathrm{RF}}\times 1$ digital signals, making FPGAs or DSPs possible implementation platforms, subject to processing-latency and control-settling measurements.

## 4. Simulation Results and Analysis

### 4.1. Simulation Setup and Baseline Schemes

To evaluate the performance of the proposed quantization-aware hybrid receiver, Monte Carlo simulations were conducted for a narrowband receiver equipped with an N=64 element half-wavelength ULA. The array is configured in a partially connected structure with $N_{\mathrm{RF}}=8$ RF chains, corresponding to M=8 antennas per subarray. The desired signal impinges from spatial direction $\theta_{s}=10^{{}^{\circ}}$ ($u_{s}=\sin10^{{}^{\circ}}\approx 0.1736$), while $N_{J}=3$ uncoordinated interference sources are located at angles $\theta_{J}=\left[-40^{{}^{\circ}},20^{{}^{\circ}},55^{{}^{\circ}}\right]$.

The input SNR is fixed at 5 dB, and the default INR is set to 25 dB per interference source. The spatial search grid spans $\left[-90^{{}^{\circ}},90^{{}^{\circ}}\right]$ with a $1^{{}^{\circ}}$ resolution, corresponding to G=181 grid points. In the default configuration, the number of sensing configurations is L=20, with $K_{l}=400$ snapshots per sensing state and $K_{d}=400$ snapshots for data reception. Both the ADC resolution and phase-shifter resolution are set to 6 bits by default. All performance curves are averaged over 200 independent Monte Carlo realizations. The default simulation parameters are summarized in Table 1.

The evaluation is a normalized narrowband baseband Monte Carlo study of the signal/covariance model in Section 2 and the three stages in Algorithm 1. Sensing covariances are corrected and fitted first; the resulting interference estimate configures the analog weights, followed by loaded MVDR processing of the data-stage covariance. Output SINR is evaluated using the desired-signal and interference-plus-noise terms in Equation (19). The simulations represent statistical quantization distortion through AQNM, not a converter transfer curve, full-scale overload test, or hardware timing measurement.

For comparison, the proposed receiver is evaluated against four benchmark schemes: fully digital MVDR (FD-MVDR), which uses dedicated RF chains and high-resolution ADCs at every antenna element to provide a fully digital performance reference [17]; orthogonal matching pursuit hybrid beamforming (OMP-HBF), which selects analog combining vectors from a steering dictionary via greedy matching pursuit [8]; phase-extraction hybrid MVDR (PE-HMVDR), which computes unconstrained digital MVDR weights and extracts their phase angles to configure phase shifters and desired-signal beam-steering (DS-BF), which steers analog beams purely toward the desired signal direction $u_{s}$ and relies entirely on digital MVDR processing for interference mitigation [28].

### 4.2. Spatial Sensing and Covariance Reconstruction Accuracy

**Figure 2:**
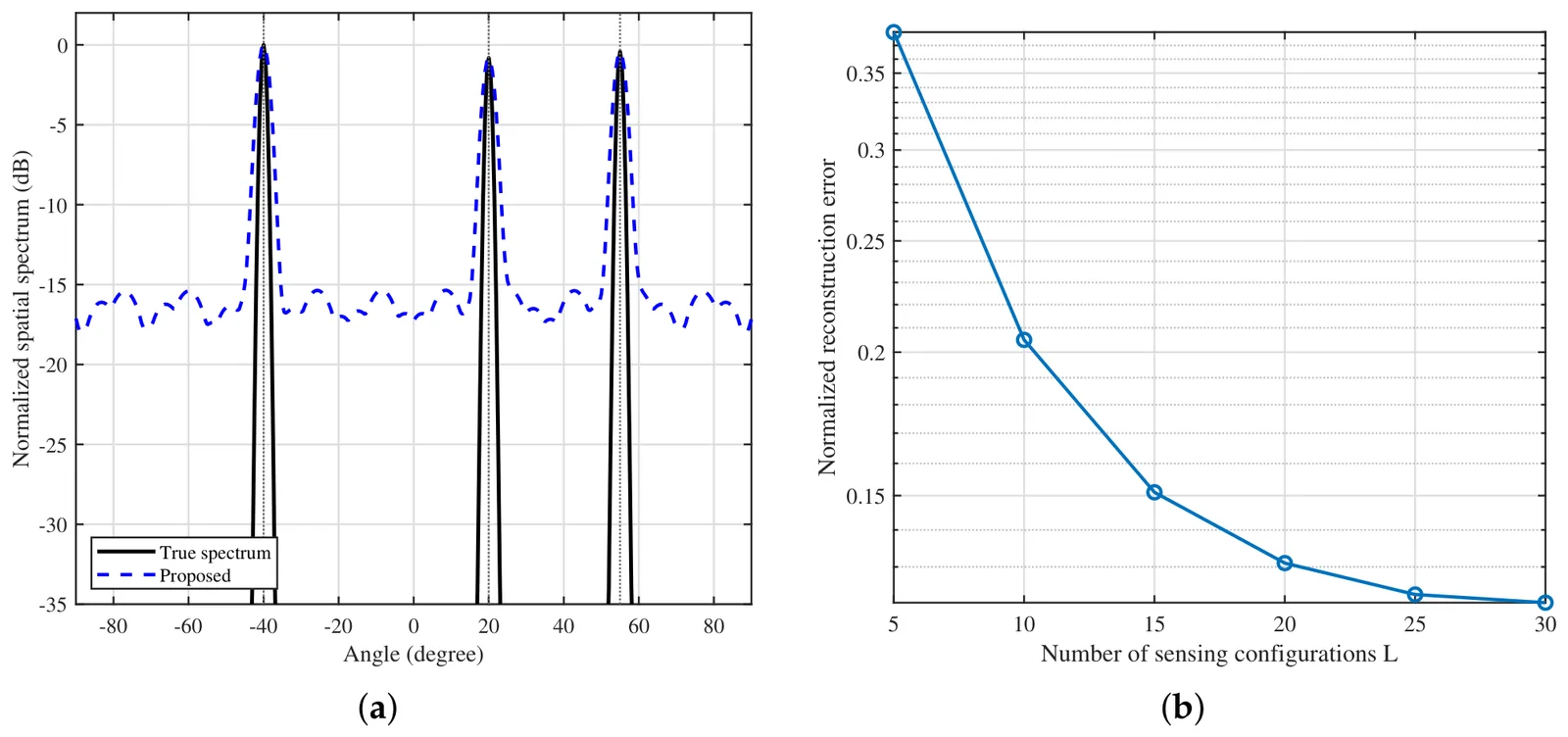
Spatial sensing performance: (**a**) Normalized angular power spectrum reconstructed by the proposed FISTA sparse fitting method; (**b**) Normalized covariance reconstruction error $\epsilon_{R}$ versus the number of sensing configurations *L*.

Figure 2 evaluates the spatial sensing performance of the proposed framework. In Figure 2a, the normalized spatial spectrum exhibits sharp, distinct peaks aligned precisely with the true interference directions at $-40^{{}^{\circ}}$, $20^{{}^{\circ}}$, and $55^{{}^{\circ}}$, while leaving the protected sector around $\theta_{s}=10^{{}^{\circ}}$ free from false detections. The minor peak broadening reflects the intrinsic trade-off of compressed dimensional sensing, but the underlying interference subspace is captured with high fidelity.

Figure 2b plots the normalized covariance reconstruction error $\epsilon_{R}={∥{\hat{R}}_{J}-R_{J}∥}_{F}/{∥R_{J}∥}_{F}$ as a function of the number of sensing configurations *L*. Increasing *L* from 5 to 15 drastically reduces the estimation error as additional random projections eliminate measurement ambiguity across subarrays. Beyond L=15, gains diminish. Finite-sample variability and regularization can leave a residual error; off-grid mismatch is not an explanation for the default on-grid directions. With $K_{l}$ fixed, increasing *L* also increases the total sample count $LK_{l}$. The choice L=20 balances sensing overhead and reconstruction accuracy in this experiment.

### 4.3. Anti-Interference Performance Under Diverse Operating Conditions

**Figure 3:**
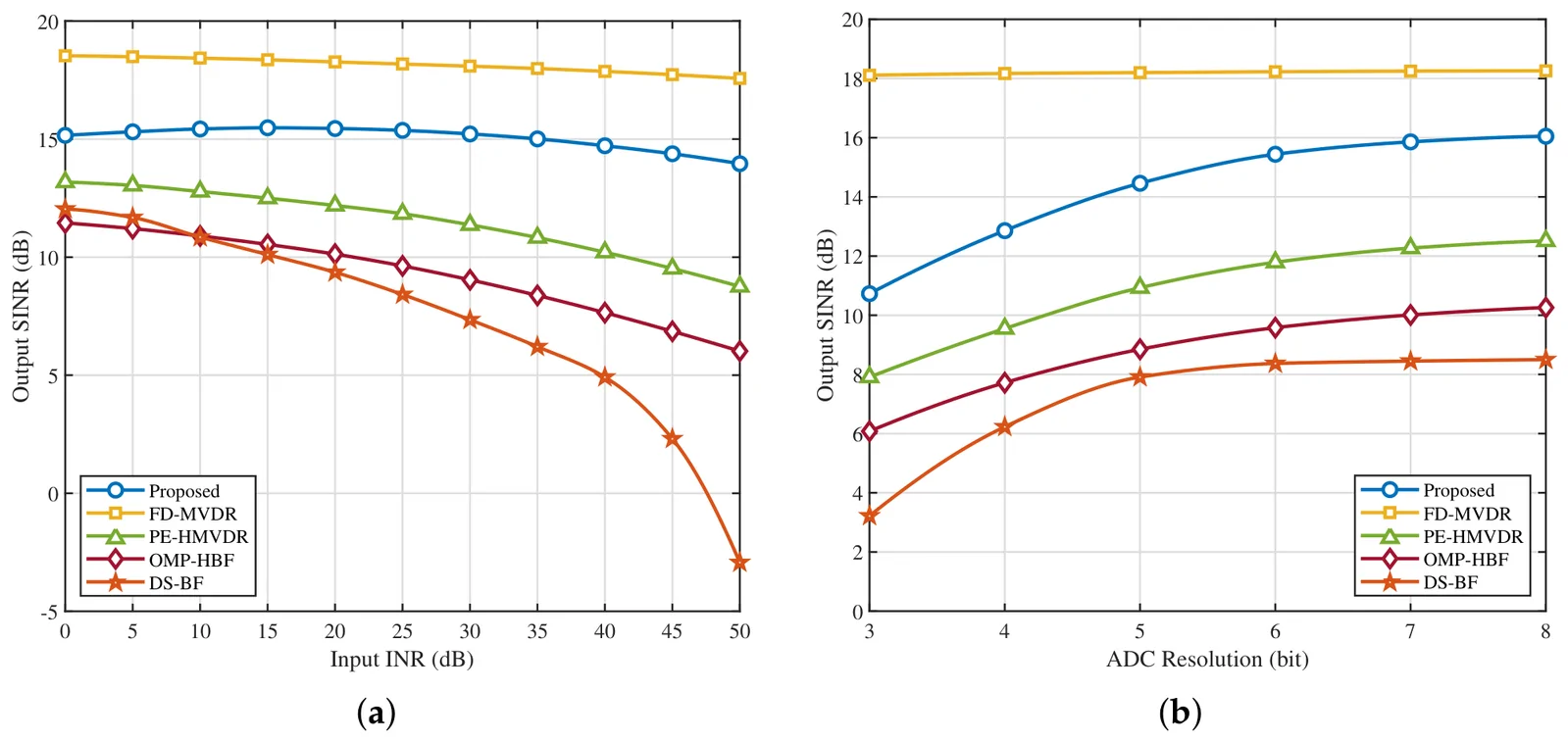
Output SINR performance under varied signal and converter conditions: (**a**) Output SINR versus input INR; (**b**) Output SINR versus ADC resolution *b*.

Figure 3 evaluates the output SINR under varying interference levels and ADC quantization bit depths. In Figure 3a, the output SINR is plotted against the input INR across 0 dB to 50 dB. The proposed receiver remains below the fully digital reference (FD-MVDR), while retaining an advantage over the plotted hybrid baselines across the evaluated INR range. In sharp contrast, the conventional DS-BF baseline collapses at high INRs because strong interference is insufficiently attenuated by the analog front-end, increasing the interference-dependent AQNM distortion term. Although OMP-HBF and PE-HMVDR incorporate interference awareness, their heuristic phase projections fail to form deep spatial nulls under hardware constraints, leading to noticeable performance degradation as INR grows.

Figure 3b examines the impact of ADC resolution from 3 to 8 bit. The proposed receiver achieves superior performance across the entire resolution range, demonstrating remarkable robustness even with coarse 3-bit converters. This resilience stems from the combined effect of AQNM-based bias removal during sensing and pre-quantization analog spatial nulling. Without analog interference attenuation, DS-BF suffers severe performance loss in the low-bit regime, underscoring that baseband suppression must contend with quantization distortion generated before digital combining.

The diminishing ADC-resolution gain is consistent with $\rho_{b}$ decreasing approximately as $2^{-2b}$ in the adopted model. Once the quantization term is small relative to residual interference, noise and estimation error, adding bits has less effect. A shallow SINR trend versus INR describes the plotted receiver performance; it is not evidence that an actual ADC avoids clipping.

**Figure 4:**
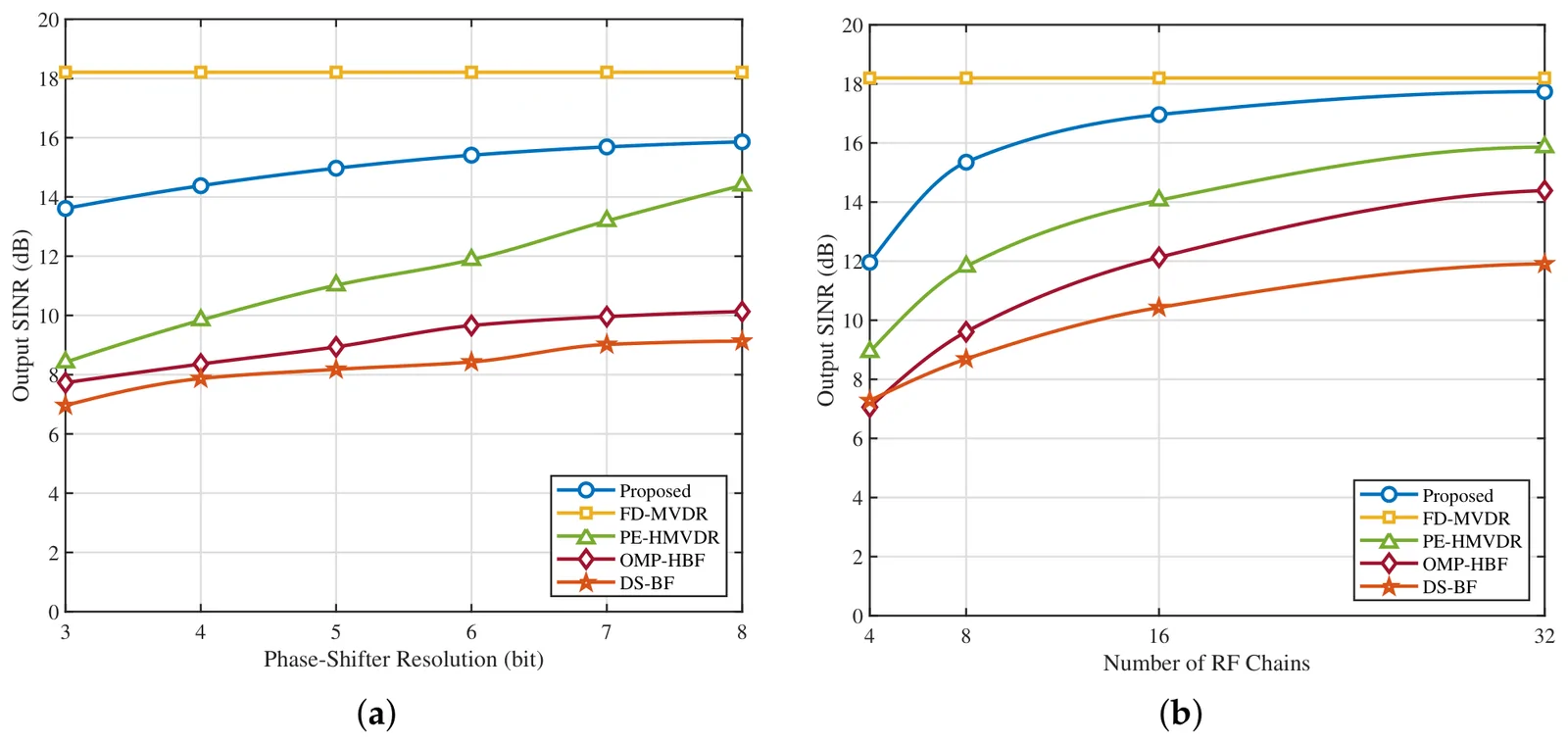
Output SINR performance under hardware constraints: (**a**) Output SINR versus phase-shifter resolution $B_{\mathrm{PS}}$; (**b**) Output SINR versus the number of RF chains $N_{\mathrm{RF}}$ (N=64).

Figure 4 illustrates the influence of phase-shifter resolution and the number of RF chains. In Figure 4a, the proposed manifold-based beamformer improves as phase resolution increases from 3 to 8 bits, with diminishing gains at finer resolution. Because Riemannian gradient descent optimizes directly on the continuous constant-modulus manifold M, the continuous solution satisfies the constant-modulus constraint. Nearest-state projection has an angular error at most $\pi /2^{B_{\mathrm{PS}}}$, which decreases with resolution; the resulting null-depth loss need not be small at coarse resolution. In contrast, PE-HMVDR truncates unconstrained weights with non-uniform amplitudes, introducing substantial approximation errors that degrade null depth when phase quantization is coarse.

Figure 4b plots the output SINR across different numbers of RF chains $N_{\mathrm{RF}}\in \left\{4,8,16,32\right\}$ for a fixed array aperture of N=64. Increasing $N_{\mathrm{RF}}$ improves output SINR for all hybrid schemes by providing higher baseband spatial degrees of freedom. Notably, the performance advantage of our proposed method is most pronounced in the highly constrained regime ($N_{\mathrm{RF}}\leq 8$). For three independent interferers, four effective dimensions can in principle support three null constraints and a desired-signal constraint. The remaining losses depend on analog compression, quantization and covariance errors. Increasing $N_{\mathrm{RF}}$ also reduces subarray size *M*, so the observed trend should not be generalized as an unconditional monotonic guarantee.

**Figure 5:**
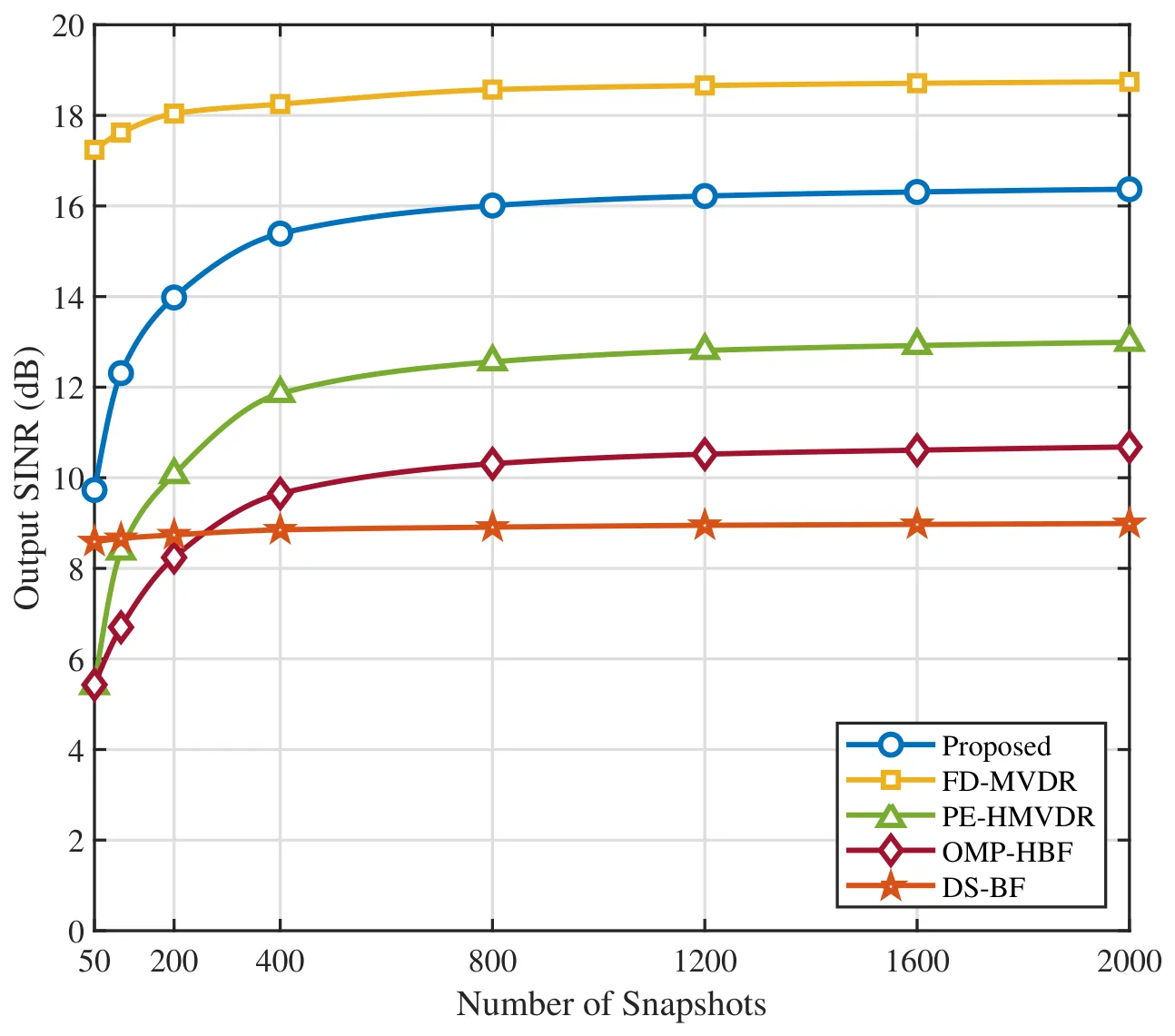
Output SINR versus number of snapshots $K_{l}=K_{d}$.

Figure 5 illustrates the output SINR as a function of snapshot count ($K_{l}=K_{d}$ varied from 50 to 2000). The curve continues to improve beyond 200 snapshots, with smaller gains at large sample counts. This behavior is consistent with decreasing covariance variability, while regularization and hardware constraints can leave a residual gap. The receiver uses the sparse covariance fitting formulation, which exploits the intrinsic low-rank structure of spatial emitters, and the diagonally loaded digital R-MVDR stage, which improves covariance conditioning.

### 4.4. Ablation Studies and Component Contribution Analysis

To isolate the individual performance gains contributed by specific algorithmic components, two ablation variants were evaluated: the variant without AQNM correction (Proposed w/o AQNM), where the dequantization step (22) is omitted and uncorrected sample covariance matrices are fed directly into sparse recovery and the variant without spatial sensing (Proposed w/o Sensing), where multi-configuration sensing is bypassed and the analog combiner is steered solely toward the desired signal direction without analog interference nulling.

**Figure 6:**
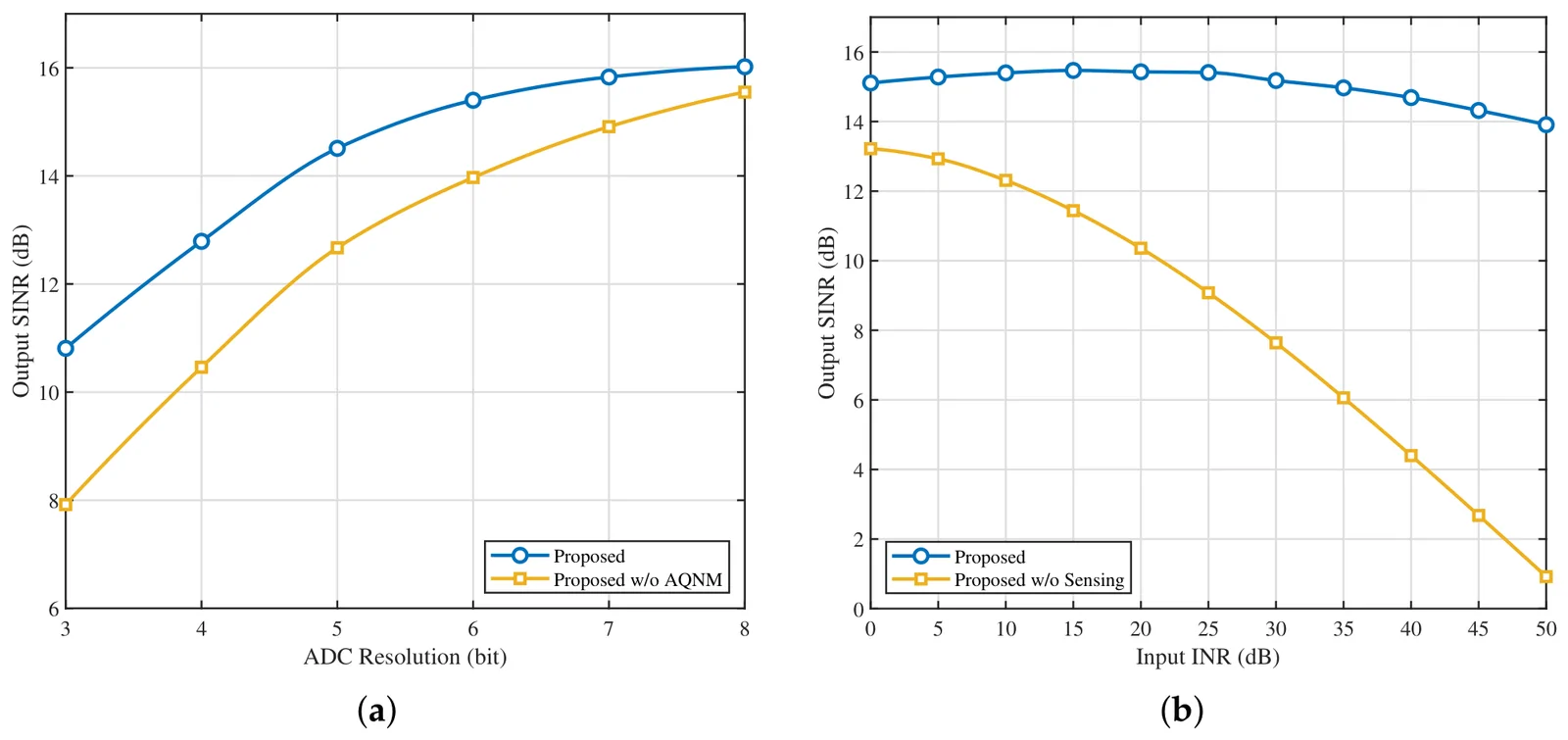
Ablation studies isolating key algorithmic components: (**a**) Output SINR versus ADC resolution with and without AQNM-based covariance correction; (**b**) Output SINR versus input INR with and without multi-configuration spatial sensing.

Figure 6 summarizes the ablation results. In Figure 6a, omitting the AQNM correction results in severe SINR degradation in the low-bit regime (b=3–4 bits), where coarse quantization distortion heavily corrupts sample covariance entries. Applying the AQNM correction compensates for the modeled covariance bias. As ADC resolution increases, the correction becomes smaller and the curves approach each other. This trend illustrates the correction within the adopted approximation and does not validate an exact inverse for physical quantization.

Figure 6b highlights the critical importance of interference spatial sensing as input INR increases. While both variants perform similarly at low INRs where thermal noise dominates, the variant without spatial sensing undergoes severe performance degradation for INR≥15 dB. Without spatial sensing, the analog combiner cannot establish nulls toward interferers, leaving more interference power at the quantizer input and increasing modeled distortion. This demonstrates that multi-configuration spatial sensing and pre-quantization nulling are essential for maintaining anti-interference capability in strong interference environments.

## 5. Discussion

The numerical evaluations confirm that the proposed quantization-aware hybrid receiver effectively resolves the tight coupling between strong directional interference, low ADC bit depth, and finite phase-shifter precision. Compared with the DS-BF baseline, whose analog weights use the desired direction without estimated interference directions, the proposed framework establishes a coordinated three-stage pipeline. By embedding the AQNM inverse correction directly into the sparse covariance fitting problem, the spatial sensing stage reconstructs the antenna-domain interference distribution with high fidelity from low-dimensional, coarsely quantized observations. Direct optimization on the constant-modulus manifold seeks to suppress dominant interferers before quantization; it does not establish a global optimum or the physical ADC overload margin. In addition, the quantized-domain robust MVDR baseband combiner uses estimated quantized-domain statistics and diagonal loading to suppress residual interference while improving conditioning.

In terms of practical deployment, the theoretical derivations and simulations in this study are based on a narrowband ULA geometry and assume stationary interference during each sensing block. In practical scenarios, array calibration errors (such as gain/phase imbalances and mutual coupling), wideband beam squint, and fast-moving interference sources present additional operational challenges. Furthermore, the scalar-gain and diagonal-distortion AQNM is an approximation, particularly at coarse bit depths. Hardware overload, correlated distortion and AGC transients may depart from this model. During initial random sensing, nulls are not yet available; practical acquisition, therefore, requires gain control or other blocker protection. Extending this framework to wideband OFDM architectures, 1-bit Bussgang receivers, and non-stationary dynamic tracking constitutes valuable directions for future investigation.

Table 2 compares recent studies by architecture, available interference information, quantization treatment and evaluation metric. Their BER, analog-suppression and spectral-efficiency results are not directly interchangeable with output SINR; the same-scenario quantitative comparisons are those in Section 4.

For component-level context, Hong et al. demonstrate an eight-element 8–16 GHz phased-array transceiver with calibrated 7-bit phase resolution [29]. Separately, a fabricated 4-bit, 500 MS/s flash ADC in 65 nm CMOS demonstrates a relevant conversion scale [30]. These are separate hardware examples, not a prototype tested with this algorithm. Integration requires downconversion, synchronized I/Q sampling, AGC and calibration. RF carrier frequency and baseband/IF sampling rate are distinct; the normalized narrowband model establishes neither a physical sample rate nor compliance with a particular wireless standard.

## 6. Conclusions

This paper addressed the problem of directional interference suppression in partially connected hybrid array receivers operating with low-resolution ADCs and discrete-phase phase shifters. A three-stage quantization-aware anti-interference framework was developed. First, multi-configuration sensing was paired with AQNM covariance correction and FISTA-based nonnegative sparse covariance fitting to reconstruct the full-dimensional interference spatial covariance from compressed, quantized measurements. Second, a constant-modulus manifold optimization scheme using Riemannian gradient descent was introduced to design subarray analog combiners that seek to improve local SINR, suppressing strong interference before ADC conversion and reducing pre-quantization interference power. Third, a diagonally loaded robust MVDR combiner was implemented in the quantized RF domain to mitigate residual interference. Simulation results demonstrated that the proposed receiver consistently outperforms conventional hybrid beamforming baselines across wide ranges of INR, ADC bit depths, phase quantization resolutions, RF chain counts, and snapshot numbers, offering an effective, hardware-efficient solution for contested large-scale array systems.

## Figures and Tables

**Table 1:** Default Simulation Parameters.

Parameter	Default Value
Number of antenna elements *N*	64
Number of RF chains $N_{\mathrm{RF}}$	8
Subarray size $M=N/N_{\mathrm{RF}}$	8
Antenna element spacing *d*	λ/2
Desired signal direction $\theta_{s}$	$10^{{}^{\circ}}$
Interference directions $\theta_{J}$	$\left[-40^{{}^{\circ}},\;20^{{}^{\circ}},\;55^{{}^{\circ}}\right]$
Input SNR	5 dB
Input INR	25 dB
ADC resolution *b*	6 bits
Phase-shifter resolution $B_{\mathrm{PS}}$	6 bits
Angular grid size *G*	181
Number of sensing configurations *L*	20
Sensing snapshots per configuration $K_{l}$	400
Data reception snapshots $K_{d}$	400
Diagonal loading factor $\kappa_{a},\kappa_{d}$	0.05

**Table 2:** Comparison with related receiver studies. All listed receiver results are simulation-based. The table compares formulation and validation scope; numerical performance is not ranked across different signal models and metrics.

Study	Analog Architecture	Sensing and Quantization Treatment	Main Evaluation
Beam-slicing [24]	Local square spatial transforms; one RF path per antenna	Fixed transform and digital SNIPS/CHOPS; covariance/channel estimation neglects quantization distortion, while detection uses Bussgang analysis	BER and served-user fraction
HERMIT [25]	Clusterwise square analog baseband transform; finite alphabets	Assumed channel/power knowledge; adaptive analog mitigation and Bussgang-aware digital equalization	BER under low-bit ADCs
Wu et al. [26]	Hybrid analog subarrays; phase-only control	Two-stage ESPRIT; MM-based analog nulling and nearest-state phase projection	Analog suppression, AoA error and convergence
Ma et al. [23]	Fully connected hybrid MIMO-OFDM with oversampling	Joint analog/digital combining with temporal and spatial quantization-distortion covariance modeling	Spectral and energy efficiency
This work	Partially connected *N*-to-$N_{\mathrm{RF}}$ compression; discrete phase control	AQNM-corrected multi-configuration sparse covariance fitting; subarray manifold optimization; loaded quantized-domain MVDR	Output SINR and sensing accuracy

## Data Availability

The original contributions presented in this study are included in the article. Further inquiries can be directed to the corresponding author.
